# Mitochondria-Targeted Protective Compounds in Parkinson's and Alzheimer's Diseases

**DOI:** 10.1155/2015/408927

**Published:** 2015-04-29

**Authors:** Carlos Fernández-Moriano, Elena González-Burgos, M. Pilar Gómez-Serranillos

**Affiliations:** Department of Pharmacology, Faculty of Pharmacy, University Complutense of Madrid, 28040 Madrid, Spain

## Abstract

Mitochondria are cytoplasmic organelles that regulate both metabolic and apoptotic signaling pathways; their most highlighted functions include cellular energy generation in the form of adenosine triphosphate (ATP), regulation of cellular calcium homeostasis, balance between ROS production and detoxification, mediation of apoptosis cell death, and synthesis and metabolism of various key molecules. Consistent evidence suggests that mitochondrial failure is associated with early events in the pathogenesis of ageing-related neurodegenerative disorders including Parkinson's disease and Alzheimer's disease. Mitochondria-targeted protective compounds that prevent or minimize mitochondrial dysfunction constitute potential therapeutic strategies in the prevention and treatment of these central nervous system diseases. This paper provides an overview of the involvement of mitochondrial dysfunction in Parkinson's and Alzheimer's diseases, with particular attention to *in vitro* and *in vivo* studies on promising endogenous and exogenous mitochondria-targeted protective compounds.

## 1. Introduction

Mitochondria are spherical cytoplasmic organelles with a symbiotic origin that are present in all eukaryotic cells. Structurally, mitochondria consist of two compositions and functionally different phospholipid membranes referred to as the outer membrane and the inner membrane and two aqueous compartments, the intermembrane space and the mitochondrial matrix. The outer membrane encloses the entire structure; it has higher content in lipids (over 60%) and it contains porins and a large multiprotein translocase complex allowing the passage to ions and larger molecules. The inner membrane surrounds the mitochondrial matrix and it invaginates to form cristae that increase total surface area. In addition, the inner membrane has lipid content over 20% and it is only permeable to small uncharged molecules. Both membranes are separated by the aqueous compartment intermembrane space, located between them [1, 2]. Moreover, mitochondria contain their own DNA (mDNA) held in the mitochondrial matrix; the human mDNA is a double-stranded circular genome made up of 16,569 base pairs of DNA that encodes 13 proteins, 22 transfer RNAs (tRNAs), and 2 ribosomal RNAs (rRNAs) [3]. Functionally, mitochondria play a vital role in regulating both metabolic and apoptotic signaling pathways. Their main function is to produce energy as adenosine triphosphate (ATP) at the mitochondrial electron transport chain (ETC) in the inner membrane, through the cellular process of oxidative phosphorylation (OXPHOS). The mitochondrial ETC consists of four integral membrane oxidation-reduction electron and proton pump protein complexes (complex I, NADH:ubiquinone oxidoreductase; complex II, succinate dehydrogenase; complex III, ubiquinone-cytochrome *c* oxidoreductase; complex IV, cytochrome *c* oxidase) and an ATP synthase (complex V) which catalyzes ADP conversion to form ATP [4]. In addition, mitochondria participate in other series of functions, including regulation of cellular calcium homeostasis, balance between ROS production and detoxification (i.e., superoxide anion (O_2_^•−^) and the highly reactive hydroxyl radical (^•^OH)), mediation of the process of programmed cell death (apoptosis), and synthesis and metabolism of endogenous compounds such as steroids, heme groups, and fatty acids [5].

Consistent evidence suggests that mitochondrial failure is associated with early events in the pathogenesis of ageing-related neurodegenerative disorders including Parkinson's disease and Alzheimer's disease. Mitochondria-targeted protective compounds that prevent or minimize mitochondrial dysfunction constitute potential therapeutic strategies in the prevention and treatment of these central nervous system diseases [6, 7]. This paper provides an overview of the involvement of mitochondrial dysfunction in Parkinson's and Alzheimer's diseases, with particular attention to* in vitro* and* in vivo* studies on promising endogenous and exogenous mitochondria-targeted protective compounds.

## 2. Parkinson's Disease and Mitochondria-Targeted Protective Compounds

### 2.1. Parkinson's Disease (PD)

Parkinson's disease is a chronic progressive disorder characterized pathologically by the loss of dopaminergic neurons located in the substantia nigra pars compacta, and, to a lesser extent, in putamen, caudate, and globus pallidus and by the formation of intracellular protein inclusions of mainly alpha-synuclein (named as Lewy bodies) in the remaining neurons [8, 9]. The first clinical description was published in 1817 by the English physician Dr. Parkinson in his work “An Essay on the Shaking Palsy” [10]. Parkinson's disease is the second most common neurodegenerative disorder after Alzheimer's disease which affects more than 6.3 million people over usually the age of 60 worldwide. Regarding epidemiology, this age-related central nervous system disease appears to be slightly more common in whites than blacks and Asian people, in men than in women, and in some geographical regions (i.e., China, India, and USA) [11–13]. The most relevant clinical features include tremor, bradykinesia, rigidity, and dystonia; however, in addition to these characteristic motor signs and symptoms, neuropsychiatric and other nonmotor manifestations such as depression, cognitive impairment, anxiety, and psychosis have been also described [8, 9, 14]. Although the exact causal factors of Parkinson's disease remain unknown, several research studies point to specific genetic mutations and environmental factors [15, 16]. It has been estimated that around 5–10 in every 100 people suffering from Parkinson's disease are associated with gene mutations. Scientifics have identified at least 13 gene mutations, among which one could highlight those in the genes SNCA (synuclein, alpha non-A4 component of amyloid precursor), PARK2 (Parkinson's disease autosomal recessive, juvenile 2), PARK7 (Parkinson's disease autosomal recessive, early-onset 7), PINK1 (PTEN-induced putative kinase 1), and LRRK2 (leucine-rich repeat kinase 2) [15]. The SNCA gene encodes for the protein alpha-synuclein, which is a key component of Lewy bodies; the PARK2 gene encodes for the E3 ubiquitin ligase parkin, which is implied in mitochondrial maintenance; the PARK7 gene encodes for the antioxidant protein DJ-1; PINK 1 gene encodes for a serine/threonine-protein kinase with a protective mitochondrial role. Alterations of SNCA, PARK2, PARK7, and PINK1 genes are involved in the early-onset Parkinson's disease (this is diagnosed before being 50 years old) [17–20]. The LRRK2 gene, which encodes for the protein dardarin, has been associated with the late-onset Parkinson's disease [21]. The rest, around 95%, of diagnosed Parkinson's disease cases are sporadic, in which environmental factors such as pesticides and dietary factors, among others, seem to play a crucial role. Researchers have identified several common pesticides that their exposure may increase the risk of developing Parkinson's disease among which rotenone, paraquat, dithiocarbamates (i.e., maneb, ziram), pyrethroids (i.e., deltamethrin), organochlorine (dieldrin), imidazoles (i.e., triflumizole, benomyl), and 2,2-dicarboximides (i.e., folpet, aptan) are included [22, 23]. Regarding dietary factors, both dietary patterns or/and dietary nutrients that may protect or may increase against to suffer from Parkinson's disease have been reported. As an example, in a case control study performed during ten years for establishing the influence of minerals, vitamins, and fats in the etiology of Parkinson's disease, an association between a high intake of a combination of iron and manganese and the development of Parkinson's disease was found [24]. On the other hand, a large prospective study performed over fifteen years with 49 692 men and 81 676 women revealed that the high intake of fruit, vegetables, legumes, whole grains, nuts, fish, and poultry, the low intake of saturated fat, and the moderate intake of alcohol are protective dietary patterns against Parkinson's disease [25].

#### 2.1.1. Mitochondrial Dysfunction in Familial PD

**Figure 1:**
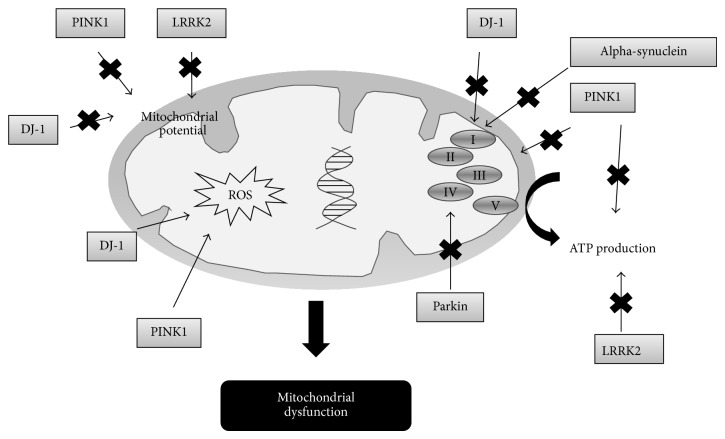
The role of gene products in Parkinson's disease.

As we have previously commented, around 5–10% of Parkinson's disease cases involve gene products. Mutations in ATP13A2 (PARK9), DJ-1 (PARK7), parkin (PARK2), and PTEN-induced putative kinase 1 (PINK1) (PARK6) are associated with autosomal recessive PD and mutations in *α*-synuclein gene and leucine-rich repeat kinase 2 gene (LRRK2) are implicated in autosomal dominant PD (see Figure 1) [16].

Mutations in the ATP13A2 gene (PARK9), encoding for a lysosomal type 5P-type ATPase, cause a hereditary rare juvenile onset autosomal recessive Parkinsonism with dementia named as Kufor-Rakeb syndrome. This particular Parkinson's form, characterized by supranuclear gaze palsy, dystonia, pyramidal signs, and cognitive impairment, was first evidenced in 2006 in members of a nonconsanguineous Chilean family. The neuronal damage associated with mutations in this gene is related to alterations in mitochondria and lysosomes functions and divalent cation regulation [26–28].

DJ-1 mutations on chromosome 1p36 cause autosomal recessive early-onset PD and its pathological mechanism seems to be linked with mitochondrial fragmentation and mitochondrial structural damage and consequently defects in the mitochondrial function of dopaminergic cells [29, 30].

Mutations in the parkin gene product, which is an ubiquitin ligase, lead to an early-onset familial Parkinson's disease and its first description dates in the year 1998. Experimental studies have determined that the pathology of parkin is associated with alterations in the mitochondrial recognition, transportation, and ubiquitination and with mitophagy impairment [31, 32].

Mutations in the mitochondrial serine/threonine-protein kinase PINK1 result in alterations in the mitochondrial morphology and function (defects in complex I activity) and they are strongly associated with a form of autosomal recessive early-onset Parkinson's disease [33, 34].

Mutations in the protein *α*-synuclein, which is the main component of Lewy bodies (it represents 1% of total cytosolic protein of brain cells), have been reported to play a key role in the pathogenesis of autosomal dominant early-onset Parkinson's disease. Particularly, two mutations in the alpha-synuclein gene (A30P and A53T) have been identified which lead to the formation of pathogenic pore-like annular and tubular protofibrils. These mutations inhibit the activity of complex I and induce mitochondrial fragmentation, causing mitochondrial dysfunction [35, 36].

Mutations in the gene encoding leucine-rich repeat kinase 2 (LRRK2) are related to autosomal dominant Parkinson's disease form. The most common mutation is G2019S that accounts for 5-6% of familial cases of Parkinson's disease. Experimental studies have identified different pathogenic mechanisms for altered LRRK2 that involve inflammation processes, oxidative stress, and mitochondrial dysfunction, among others. Focusing on this last pathogenic mechanism, mutations in LRRK2 cause mitochondrial fragmentation and a downregulation in mitochondrial homeostasis (reduction in mitochondrial membrane potential and ATP production) [37, 38].

#### 2.1.2. Mitochondrial Dysfunction in Sporadic PD

Around 95% of diagnosed Parkinson's disease cases are sporadic. One of the proposed mechanisms for the dopaminergic neurons degeneration in sporadic Parkinson's disease cases is related to an excessive production of reactive oxygen species (ROS) that leads to oxidative stress situation. An excess of ROS causes the oxidative modification of macromolecules (lipids, proteins, and DNA) leading to cell damage and even cell death. The pathological effect of ROS is also involved in a reduction of ATP (adenosine triphosphate) production, in an increase of iron levels, and in an increase of intracellular calcium levels and alterations in mitochondrial respiratory chain complexes function. In addition to oxidative stress mechanism, protein misfolding, aggregation, and deposition have been reported as other common pathological mechanisms in Parkinson's disease. A dysfunction in the ubiquitin-proteasome-system (UPS) and the autophagy-lysosomal pathway (ALP) as evidenced in a reduction of proteasome and autophagy activities and in postmortem brains of patients suffering from this neurodegenerative disease has been demonstrated [39–43].

#### 2.1.3. Postmortem PD Brain Tissues, Experimental Models, and Cell-Based Models

Many evidences from postmortem PD brain tissues, experimental models, and cell-based models have demonstrated the involvement of mitochondria dysfunction in the pathogenesis of both familial and sporadic Parkinson's disease.

The first evidence of the relationship between mitochondria and Parkinson's disease dates from the second half of the twentieth century when the postmortem brain analysis of some drug abusers of intravenous 1-methyl-4-phenyl-1,2,3,6-tetrahydropyridine (MPTP), who have developed a progressive and irreversible parkinsonism, revealed a significant nigrostriatal degeneration. MPTP easily passes through the blood-brain barrier; it is oxidized and transformed into 1-methyl-4-phenylpyridinium (MPP+) and within neurons MPP+ inhibits the complex I (NADH-quinone oxidoreductase) of the electron transport chain, resulting in an enhanced reactive oxygen species (ROS) generation (i.e., hydroxyl radicals, superoxide anion radical) and a decrease in energy supply (ATP production) [44]. Many lines of evidence have further demonstrated complex I deficiency or impairment in the cortical brain tissue, frontal cortex, striatum, skeletal muscle, and platelets of patients with Parkinson's disease [40, 45, 46]. In addition to complex I, other studies have reported that a deficiency in the activity of complex II (succinate ubiquinone oxidoreductase) and complex III (ubiquinol-cytochrome C oxidoreductase) is also associated with the pathogenesis of Parkinson's disease. Complex III inhibition, as what happens with complex I, causes an overproduction of ROS, leading to oxidation of lipids, proteins, and DNA and it finally triggers to cell death [47, 48]. Moreover, ROS mediates the mitochondrial-dependent apoptosis by inducing mitochondrial permeability transition, releasing of cytochrome c, activation of caspase-3 and caspase-9, translocation of Bax to mitochondria, and the activation of c-Jun N-terminal kinase (JNK) and p38 mitogen-activated protein kinase (p38 MAPK) in the cytosol [49, 50].

The neurotransmitter dopamine has been also related to the pathogenesis of Parkinson's disease.* In vitro* experimental researches on neuronal cell types and isolated brain mitochondria and* in vivo* studies using different animal models and postmortem brain studies in Parkinson's disease have demonstrated that dopamine oxidation and reactive dopamine quinone oxidation products induce mitochondrial respiration uncoupling and cause ATP levels reduction and inactivate proteasomal activity, among other effects, which contribute to mitochondrial dysfunction [51–57]. The role of tetrahydrobiopterin (BH4) in Parkinson's disease etiology is also remarkable; BH4 is an obligatory cofactor for the dopamine synthesis enzyme tyrosine hydroxylase and it is present selectively in monoaminergic neurons in the brain. It has been suggested as an endogenous molecule that contributes to the dopaminergic neurodegeneration through an inhibition of the activities of complexes I and IV of the electron transport chain (ETC), together with a release of mitochondrial cytochrome C and a reduction of mitochondrial membrane potential [58].

There are other studies which involve calcium excitotoxicity and nonexcitotoxicity related mechanisms in the etiology of Parkinson's disease. Alterations in calcium influx in neurons via L-type voltage-dependent channels and N-methyl-D-aspartate (NMDA) receptors may lead to an excitotoxic cellular calcium accumulation that can cause mitochondrial dysfunction by reducing ATP production, activating mitochondrial permeability transition, increasing ROS generation, and inducing mitochondrial-dependent apoptosis [59, 60]. Other circumstances, not ordinarily toxic, have been reported to contribute to mitochondrial dysfunction. Hence, Sheehan et al. (1997) showed using mitochondrially transformed cells (cybrids) that the capacity to sequestrate calcium was lower in patients with Parkinson's disease than in control subjects, suggesting that this homeostasis alteration could increase neurons cell death [61].

Regarding familial Parkinson's disease, mutations in several genes previously reported (Parkin, PINK1, DJ-1, *α*-synuclein, and LRRK2) which encode for mitochondrial proteins have been identified to contribute to mitochondrial dysfunction [62, 63].

There are different neurotoxins including rotenone, 1-methyl-1,2,3,6-tetrahydropyridine (MPTP), 6-hydroxydopamine (6-OHDA), and paraquat, among others, which have been extensively used as Parkinson's disease experimental models to mimic the neuropathology of this neurodegenerative disorder in both* in vitro* (i.e., human neuroblastoma SK-N-SH cells) and* in vivo* (animals models such as rats, mice) investigations and, consequently, to help establish neuroprotective strategies. Rotenone, an insecticide extracted from the roots of* Derris* spp. and* Lonchocarpus* spp. (Leguminosae family), acts by inhibiting the mitochondrial respiratory chain complex I [64]. Paraquat (1,1-dimethyl-4,4′-bipyridinium dichloride), which is a quaternary nitrogen herbicide used to control weed growth, has been reported to increase ROS generation and induce *α*-synuclein fibril formation [65]. The 1-methyl-1,2,3,6-tetrahydropyridine (MPTP), a byproduct obtained during the chemical synthesis of a meperidine analog, is metabolized in the brain to the toxic compound MPP+ which inhibits complex I of the electron transport chain [44]. The catecholaminergic neurotoxin 6-hydroxydopamine (6-OHDA), via intracerebral infusion, causes the irreversible loss of nigrostriatal dopaminergic neurons by inducing ROS production and inhibiting complex I and complex IV of the electron transport chain [66].

### 2.2. Mitochondria-Targeted Protective Compounds in PD

**Figure 2:**
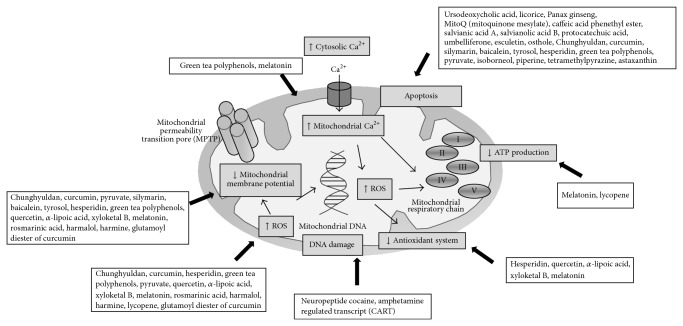
Mechanisms of mitochondrial dysfunction and mitochondria-targeted drugs that have produced beneficial effect in PD models.

Endogenous and exogenous compounds are in continuing investigation as mitochondria-targeted agents to prevent or treat Parkinson's disease (Figure 2).  Table 1 reports compounds that have been demonstrated to be promising agents in the protection of mitochondrial dysfunction in different Parkinson's disease models. Hence, among the endogenous compounds investigated so far, the hormone melatonin, the neuropeptide cocaine, and amphetamine regulated transcript (CART), the ursodeoxycholic acid, the mitoQ (mitoquinone mesylate), and the *α*-lipoic acid can be highlighted. The hormone melatonin has been shown to exert* in vivo* mitochondrial protective action in MPTP-induced mice model, 6-OHDA rat model, and rotenone-induced rat model by maintaining mitochondrial membrane potential, increasing antioxidant enzymatic (i.e., SOD, CAT) and nonenzymatic levels (i.e., glutathione), inhibiting ROS overproduction, increasing ATP production, decreasing calcium concentration levels, and enhancing mitochondrial complex I activity [67–71]. The neuropeptide cocaine and amphetamine regulated transcript (CART) protected mitochondrial DNA and cellular proteins and lipids of human neuroblastoma SH-SY5Y cells, HEK293 cells, and cultures of cortical and hippocampal neurons exposed to hydrogen peroxide [72]. The ursodeoxycholic acid (one of the secondary bile acids) and the mitoQ (mitoquinone mesylate) acted as antiapoptotic agent in human neuroblastoma SH-SY5Y cells treated with SNP and 6-OHDA, respectively [73, 74]. The *α*-lipoic acid has been evaluated as mitochondrial-targeted protective compound in several* in vitro* and* in vivo* Parkinson's disease models (i.e., PC12 cells, SK-N-MC cells, and rat model; toxins as MPP(+) and rotenone); this organosulfur compound derived from octanoic acid protects mitochondria by inhibiting ROS production, increasing glutathione levels, and maintaining mitochondrial membrane potential [75–78]. Pyruvate has also been demonstrated in* in vitro* studies that maintains mitochondrial membrane potential and inhibits ROS generation and nuclear translocation of NF-kappaB as well as mitochondrial apoptotic pathway [79, 80].

Several natural products from medicinal plants, both isolated compounds and extracts, have been demonstrated in* in vitro* and* in vivo* studies to exert promising mitochondrial protection. As extracts, it has been reported that berries rich in anthocyanidins and proanthocyanidins protect mitochondria from rotenone-induced changes in the respiratory chain [118]. The silymarin, which is a standardized extract of the milk thistle seeds, maintained mitochondrial integrity and function and inhibited mitochondrial apoptotic pathway in MPP(+)-induced rat model [119]. Green tea polyphenols have been also evidenced to inhibit mitochondrial apoptotic pathway (increasing Bcl2 and decreasing caspase-3 activity) and to maintain mitochondrial membrane potential, to inhibit ROS production and calcium concentration levels [120]. The licorice (root of* Glycyrrhiza glabra*) inhibited dopaminergic apoptotic cell death as evidenced in the increase in Bcl2 levels and in the decrease in Bax levels, caspase-3 activity, cytochrome c release, and JNK and MAP activities in a model of 6-OHDA-induced Parkinson's disease [121]. The water extract of* Panax ginseng* also inhibited apoptosis MPP(+)-induced in the human neuroblastoma SH-SY5Y cells by decreasing Bax levels, caspase-3 activity, and cytochrome release and increasing Bcl2 levels [122].

The herbal medicine Chunghyuldan inhibited caspase-3, ROS generation and maintained mitochondrial membrane potential in 6-OH Parkinson's disease model [123]. Among isolated natural products, highlight those with polyphenol structure. The polyphenol resveratrol has been demonstrated in* in vitro* primary fibroblasts cultures from patients with parkin mutations (PARK2) to regulate mitochondrial energy homeostasis as evidenced in the increment of complex I activity, citrate synthase activity, basal oxygen consumption, and ATP production and in the decrement of lactate content [111]. The polyphenol hesperidin inhibited mitochondrial apoptotic pathway (increased Bcl2 levels and decreased Bax, caspase-3, and caspase-9 activities and inhibited cytochrome c release), maintained mitochondrial membrane potential, inhibited ROS production, and increased glutathione levels in* in vitro* human neuroblastoma SK-N-SH cells model of rotenone-induced Parkinson's disease [98]. Quercetin rescued toxic-induced defects in mitochondria in* in vitro* and* in vivo* experiments. Quercetin inhibited ROS generation and maintained mitochondria membrane potential in rotenone-induced rat model [108]. Moreover, quercetin decreased the production of superoxide radicals and inhibited the expression of the inducible nitric oxide synthase protein expression in* in vitro* glial-neuronal system model of MPP(+)-induced Parkinson's disease [109]. The flavonoid baicalein inhibited* in vitro* apoptotic mitochondrial cell death and maintained mitochondrial integrity and function in both SH-SYTY and PC12 cells in 6-OHDA and rotenone Parkinson's disease models as evidenced in the decrease in caspase-3, caspase-7, caspase-9, and JNK activities and in the maintenance of mitochondrial membrane potential, increment of ATP content and reduction of ROS production [82–84]. The tyrosol protected CATH.a cells against MPP(+)-toxicity by inhibiting apoptotic cell death via activation of PI3K/Akt signaling pathway and by maintaining ATP production and mitochondria membrane potential [116]. The caffeic acid phenethyl ester inhibited 6-OHDA-induced mitochondrial apoptotic pathway in* in vitro* and* in vivo* models [85, 86]. The curcumin polyphenol derived from the spice turmeric acts as mitochondrial antiapoptotic agent through the inhibition of caspase-3 and caspase-9 activities and cytochrome c release and it also protects mitochondrial integrity and function via ROS production inhibition and complex I activity enhancement [89, 90]. In addition to curcumin, its synthetic pyrazole derivative compound, CNB-001, has been also studied, which avoids rotenone-induced mitochondrial damage in the human neuroblastoma SK-N-SH cells by inhibiting mitochondrial apoptotic pathway and maintaining mitochondrial structure [87, 88]. Glutamoyl diester of curcumin has also been shown to maintain mitochondrial membrane potential and to inhibit ROS production in mouse brain mitochondria induced-peroxynitrite Parkinson's disease model [96]. Salvianic acid A and salvianolic acid B isolated from* Salvia* spp. as well as protocatechuic acid afford* in vitro* protection through antiapoptotic pathway [107, 113, 114]. The flavonoid kaempferol exerts antiparkinsonian effect via autophagy [100] and rosmarinic acid maintains mitochondria protection by maintaining mitochondrial membrane potential and decreasing ROS production [112].

Other natural products with mitochondrial protective effect are the coumarins umbelliferone, esculetin, and osthole which in* in vitro* and* in vivo* Parkinson's disease models have been demonstrated to possess antiapoptotic properties on mitochondria [94, 104]. Other compounds that exert protection via inhibition of the mitochondrial apoptotic pathway are the monoterpenoid alcohol isoborneol [99], the alkaloid piperine [105], the pyrazine tetramethylpyrazine [81], and the nonprovitamin A carotenoid astaxanthin [115]. On the other hand, the *β*-carboline alkaloids harmalol and harmine maintained mitochondria membrane potential and decreased ROS generation in PC12 cells exposed to S-nitroso-N-acetyl-DL-penicillamine (SNAP) [97]. Moreover, the triterpenoid xyloketal B also maintained mitochondria membrane potential and decreased ROS generation and it increased glutathione levels in* in vitro* rat adrenal pheochromocytoma (PC12) cells and* Caenorhabditis elegans* of MPP(+)-induced PD [117]. Furthermore, the carotenoid lycopene inhibited macromolecular mitochondrial damage (lipids, DNA, and proteins), overproduction of ROS, ATP failed production, and cytochrome c release in MPP(+)-induced human neuroblastoma SK-N-SH cells and rotenone-induced rat model [101, 102].

## 3. Alzheimer's Disease and Mitochondrial-Targeted Protective Compounds

### 3.1. Alzheimer's Disease

Alzheimer's disease (AD) is a neurodegenerative disease characterized by progressive cognitive decline leading to complete need for care within several years after clinical diagnosis [124]. AD is the most common form of dementia, being the most prevalent neurodegenerative disease (followed by Parkinson's disease), and accounts for approximately 65% to 75% of all dementia cases. It has been estimated that Alzheimer's disease affects over 44 million people worldwide, mainly after the age of 65 years [125, 126]. The incidence of AD augments with age in an exponential manner and its prevalence increases from 3% among individuals aged 65–74 to almost 50% among those 85 or older; these numbers can be translated to the extremely high health care costs that AD represents [127]. In addition, because of the aging of the population, it is expected that the prevalence will quadruple by 2050, which means 1 in 85 persons worldwide will be living with the disease [128].

AD is a progressive neurodegenerative disease with a marked late onset (late diagnosis as well) and mainly characterized by progressive decline of cognitive functions, memory, and changes in behavior and personality [129, 130]. The two major pathophysiological hallmarks that have been observed in* postmortem* brains of AD patients include extracellular *β*-amyloid protein (A*β*) deposits in the form of senile plaques and intracellular deposition of the microtubule-associated protein tau as neurofibrillary tangles, especially abundant in the regions of the brain responsible for learning and memory. These features have been linked to an abnormally enhanced neuronal loss in this condition, especially affecting cholinergic neurons and consequently leading to a reduction in the levels of the neurotransmitter acetylcholine in the hippocampus and cortex areas of brains of AD patients. Moreover, AD has also been associated with the loss of synapses, synaptic function, inflammatory responses involving glial cells, and mitochondrial abnormalities [131–133].

Considering AD pathogenesis, multiple etiological factors including genetics, environmental factors, diet, and general lifestyles have to be taken into account [134]. Most of the cases of AD are believed to be “sporadic” and their causal factors are still unknown for the vast majority of patients; on the other hand, genetic factors cause about 2% of all AD cases and include mutations in APP (A*β* protein precursor), presenilin-1 and presenilin-2 genes, and polymorphisms in apolipoprotein allele E4 [135, 136].

Due to the complex and not fully understood etiopathology of AD, no available drug has been shown to completely protect neurons in AD patients, and there is a continuous search for new compounds and therapeutic tools. There are two possible conceptual approaches to the treatment of AD. The first one is a symptomatic treatment that tries to minimize tertiary cognitive symptoms and protects from further cognitive decline; it is the most common therapeutic tendency and drugs such as tacrine, donepezil, and rivastigmine have been used with this purpose with limited efficacy. Another approach is the treatment addressed to prevent the onset of the disease by sequestering the primary progenitors or targets, to reduce the secondary pathologies of the disease, to slow disease progression, or to delay onset of disease, by preventing or attenuating neuronal damaging factors [137, 138]. With regard to this, compounds that exert activity against oxidative stress and mitochondrial dysfunction in AD (as discussed below) deserve to be considered as potential therapeutic options.

During the last two decades, consistent evidences have proposed oxidative stress as a crucial pathogenic mechanism underlying AD [139]. Oxidative stress (OS) occurs when the production of reactive oxygen species (ROS) exceeds the antioxidant enzymatic and nonenzymatic cellular mechanisms. Actually, the *β*-amyloid peptide A*β*_1–42_ (insoluble form), which forms the senile plaques, exerts neurotoxicity involving OS in AD. Particularly, this A*β*_1–42_ has the ability to produce ROS, mainly hydrogen peroxide, when it reacts with transition metal ions present in senile plaques [140]. As a result of OS, accumulated oxidative damage to lipids, proteins, and nucleic acids in* postmortem* studies of brains of patients with AD has been identified: advanced glycation end-products (AGEs), advanced lipid peroxidation end-products, nucleic acid oxidation, carbonyl-modified neurofilament protein, and free carbonyls [141]. The brain is more susceptible to OS than other organs because of a low antioxidative protection system, which allows for increased exposure of target molecules to ROS; the higher level of ROS, together with neuroinflammation and excessive glutamate levels, is proposed to contribute to neuronal damage and death in AD [142].

#### 3.1.1. Mitochondrial Dysfunction in AD

Mitochondria are the primary source of ROS, and oxidative damage to mitochondrial components precedes damage to any other cellular component during the development of neurodegenerative diseases [143]. Actually, mitochondrial dysfunction has largely been demonstrated as one of the main key cytopathologies of AD [144, 145]. Numerous evidences suggest the involvement of *β*-amyloid protein deposits in the mitochondrial dysfunction found in AD as a plausible mechanism for its neurodegenerative effects [146–148]. In support of this, it has been shown that cells depleted of endogenous mDNA lacking functional electron transport chains (ETC) are resistant to A*β* toxicity [149]; also, a reduced respiratory capacity and low cytochrome oxidase activity were found in isolated mitochondria exposed to A*β* [150, 151]; transgenic mice expressing mutant APP (amyloid protein precursor) genes exhibit mitochondrial dysfunction, and an AD transgenic mouse line presents early expression of genes encoding mitochondrial proteins and ETC subunits, as an initial cellular change in AD pathology [152].

Mitochondria have been shown to be a direct site of A*β* accumulation in AD neurons, and various experimental models of AD were used by researchers to verify the effect of that specific accumulation on cell death [153]. Actually, Manczak et al. proved an association between mutant APP derivatives (A*β* monomers and oligomers, such as A*β*_1–40_ and A*β*_1–42_) and mitochondria in cerebral cortex slices from Tg2576 mice and N2a cells expressing mutant APP. Such accumulation supposes an increase in mitochondrial ROS production together with a reduced Cyt C oxidase activity, thus relating* in vivo* oxidative stress and impaired mitochondrial metabolism to the toxic effects of A*β* peptides [154]. Further, Devi et al. demonstrated that the mitochondrial dysfunction in human AD brain is associated with the abnormal accumulation of APP across the mitochondrial import channels. In* postmortem* evaluations, it was evidenced that nonglycosylated full-length and C-terminal truncated APP had been accumulated exclusively in the protein import channel of mitochondria of AD brains (specially higher accumulation in AD-vulnerable regions, such as cortex, hippocampus, and amygdala), by forming stable complexes with the outer membrane translocase and/or the inner membrane translocase; the effect of such association could inhibit the entry of nuclear encoded Cyt C oxidase protein, thus diminishing its activity in mitochondria and increasing the levels of H_2_O_2_. The higher the level of arrested mitochondrial APP, the worse the mitochondrial dysfunction [155].

What is more, a recent study indicated that mitochondria-targeted A*β*_1–42_ accumulation is the necessary and sufficient condition for A*β*-mediated mitochondrial impairments and derived cellular death. In an* in vitro* model of mice hippocampal cell line (HT22 cells), an exogenous A*β*_1–42_ treatment caused a deleterious alteration in mitochondrial morphology and function, which was blocked by a clathrin-mediated endocytosis blocker; besides, specific mitochondria-targeted accumulation of A*β*_1–42_ in HT22 cells using a mitochondria-targeting sequence reproduced the same morphological and functional alterations of mitochondria as those observed in APP mutant mice model and the previous A*β*_1–42_-treated HT22 cells. Mitochondria-mediated apoptotic cell death was observed in both models, thus implying that no other signaling alteration induced by A*β* plays a more relevant role in cell death than its mitochondrial toxicity [156].

In general, mitochondrial dysfunction in AD is essentially characterized by diminution in complex IV activity (cytochrome c oxidase), decline in other enzymes of tricarboxylic acids cycle, and mutations to mDNA. The mechanism that underlies the complex IV defect is not clearly known, but a study on SK-N-SH cells exposed to A*β*-induced toxicity showed a decrease in mDNA encoded complex IV subunits, at both the mRNA and protein levels; this finding suggests a possible relationship between decreased complex IV activity and mDNA perturbation [157]. Results from cybrids studies also imply that AD is characterized by specific mDNA mutations that correlate with defects in certain mitochondrial respiratory complexes. These changes generate an increased production of oxidant species and free radicals, such as hydrogen peroxide. In turn, a deficiency in energy metabolism and ATP generation is a serious consequence of impaired mitochondrial function [158, 159]. In addition, deficiency in scavenging mitochondrial free radicals may similarly contribute to the excessive oxidative damage in the affected brain regions in AD. For instance, decreased mitochondrial MnSOD expression level has been found in AD patients as well as decreased Coenzyme Q in peripheral tissues and brains [160, 161]. Therefore, a relationship between the mitochondrial dysfunction and the oxidative stress situation is established.

Neurodegeneration and synaptic degradation in AD are primarily mediated by defective mitochondrial biogenesis and axonal transport of mitochondria [162]. Normal mitochondrial dynamics, an essential function in maintaining cell viability, is likewise impaired in AD. Disturbances affecting the balance of fusion and fission processes trigger serious mitochondrial changes and lead to cellular perturbations, such as apoptosis. Recent studies have found altered levels of mitochondrial fusion (including MNF-1/2 and OPA1) and fission (FIS1) proteins in AD hippocampal tissues, meaning decreased fusion and increased fission processes; mitochondrial fission protein DLP1 has also been found to be decreased in hippocampal neurons [163]. Moreover, mitochondrial calcium overload is another feature of mitochondrial dysfunction in AD; A*β* has been shown to cause calcium overload that then causes increased free radical accumulation and provokes the formation of mitochondrial transition pore (mPTP), thus leading to exacerbation of cytoplasmic calcium and eventual neuronal death [164].

Further, mitochondria play a pivotal role in aging and senescence, contributing to neural dysfunction with age. They are actually the main cellular organelle implicated in the process of neuronal apoptosis, which takes place in an excessive manner in AD brains [165]. The fact that many neurons undergo apoptosis in AD is evidenced by the presence of high levels of activated proapoptotic proteins such as caspase-3 and Bax in neurons that exhibit neurofibrillary tangle pathology [166].

Concerning AD models of study, unfortunately, there is no animal model so far that replicates all the major aspects of AD pathology and symptoms, and models based on postulated disease pathways are widely used to explore biological targets [167]. Regarding the investigation of the effects of compounds on mitochondrial dysfunction, rodent transgenic models are very common for reproducing the mitochondriopathy features in AD. For instance, an APP (amyloid precursor protein) mice transgenic model demonstrated an accelerated upregulation of the apoptotic-related factors involved in mitochondria-mediated apoptosis, such as Bax and caspase-3 [168]. Similarly, isolated mitochondria from APP_SW_ mice (expressing the Swedish familial mutation in APP gene) presented an abnormally reduced mitochondrial respiratory rate, mitochondrial membrane potential (MMP) disruption, increased ROS generation, and lower ATP levels [169]. APP/PS1 transgenic mice include mutations both in APP and in presenilin-1 genes and show similar mitochondrial characteristics [170]. Other models even express more mutations, such as 3xTg-AD mouse model that includes three mutant human genes: APPswe, presenilin-1 (PS1M146V), and tau protein (tau P301L); in this model, MMP loss and higher caspases 3 and 9 activations are observed [171].

However, most of the works assessing mitochondrial defects in AD are still performed on toxin-induced* in vitro* models. With this respect, rat primary neurons in culture exposed to A*β*_1–42_ oligomers reproduced the generation of mPTP in mitochondrial membrane with subsequent calcium overload, the MMP loss, and release of cytochrome C, thus leading to cell death via mitochondrial-mediated apoptosis [172]. Mouse neuroblastoma N2a cells cotransfected with Swedish mutant APP and Δ9 deleted presenilin-1 (N2a/Swe.Δ9) recapitulated similar loss of mitochondrial integrity and function and evidenced increased mitochondrial apoptotic pathway, with a higher Bax/Bcl2 ratio and augmented caspase-3 activity [173]. Recent studies have employed cybrid neurons resulting from incorporating platelet mitochondria from AD patients into mitochondrial DNA-depleted neuronal cells (SH-SY5Y cell line); this model demonstrates changes in length and density of mitochondria, imbalanced mitochondrial fission, and fusion dynamics (altered expression and distribution of DLP1 and Mfn2 proteins), together with reduced mitochondrial function and energy metabolism [174].

**Figure 3:**
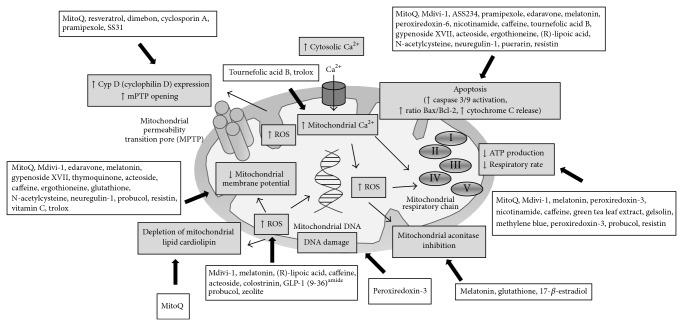
Mechanisms of mitochondrial dysfunction and mitochondria-targeted drugs that have produced beneficial effect in AD models.

Therefore, it has been suggested that a substance of exogenous or endogenous origin that is able to reverse any of the aforementioned mitochondrial deficits may facilitate a better neuronal health and then be of interest of study as a potential active compound in AD therapy [175]. In fact, mitochondrial medicine is emerging as a field of research focused on the finding of therapeutic strategies to enhance mitochondrial function in aging and in those neurodegenerative diseases in which it has been shown to be impaired [176–178]. This avenue of investigation has led to the discovery of several agents directly targeted to mitochondria that are able to delay or revert the mitochondrial impairments associated to AD; all available information on these compounds is reviewed below and collected in Table 2 and schematized in Figure 3.

### 3.2. Mitochondria-Targeted Protective Compounds in AD

#### 3.2.1. Synthetic Compounds

Several mitochondria-targeted antioxidants have been designed by conjugating the lipophilic triphenylphosphonium (TPP+) cation to an antioxidant moiety, such as coenzyme Q (CoQ), obtaining as a result compounds like MitoQ [219]. Due to its chemical nature, MitoQ takes advantage of the large MMP for reaching high concentrations in mitochondria and, unlike isolated CoQ, it is an effective antioxidant in the absence of functional ETC [220, 221]. McManus et al. demonstrated that MitoQ is effective in preventing loss of spatial memory and delaying the early neuropathology in a triple transgenic mouse model of AD; they evaluated its effect on mitochondrial deficiency and found that MitoQ avoided the MMP drop and reduced the apoptosis in cortical neurons by a decrease in caspase-3 activity [171]. Another study employed a* Caenorhabditis elegans* model overexpressing human A*β* end evidenced that MitoQ exerted protective effects on lifespan and A*β*-induced paralysis and markedly ameliorated the depletion of mitochondrial lipid cardiolipin and increased the mitochondrial ETC function by protecting complexes IV and I; however, it was not able to reduce the A*β*-induced mitochondrial DNA oxidative damage [201].

Recently, Szeto developed a series of small, cell-permeable antioxidant peptides (SS peptides) that are known to protect mitochondria from oxidative damage [222]. SS31 (H-D-Arg-Dmt-Lys-Phe-NH2) is one of them and presents a sequence motif that allows it to target mitochondria. In an A*β*_25–35_-induced AD model of mice hippocampal neurons, SS31 restored axonal transport of mitochondria and displayed promising protection and maintenance of mitochondrial function, proved by an increase in the number of healthy and intact mitochondria and a reduction in the levels of fission proteins, matrix protein, and CypD [162]. Similar results were found by Calkins et al. [211]. Manczak et al. also revealed positive effects on mitochondria for SS31; it was able to normalize the number of mitochondria and reduce the abnormal expression of peroxiredoxins and mitochondrial structural genes, that was present in an A*β*_25–35_-induced mouse N2a cells AD model [200]. Moreover, the mitochondrial division inhibitor 1 (mdivi-1) attenuated the degree of apoptosis in an A*β*-induced model of AD in BV-2 and primary microglial cells, then counteracting another pathological feature of AD, such as neuroinflammation; this effect is probably mediated through its effects on mitochondria, since Mdivi-1 reversed abnormal mitochondrial fission, MMP loss, CytC release, and caspase-3 activation [223]. Besides these actions, in a cybrid cell model, it also maintained mitochondrial integrity and function, via a reduction of mitochondrial ROS production, and increases in CytC oxidase and SOD activities and ATP levels [174]. Another chemically synthesized compound such as complex ASS234, a novel multipotent molecule that combines indolyl propargylamine and benzylpiperidine moieties, has shown protective activities in an A*β*_1–42_-induced SH-SY5Y neuroblastoma cells model of AD; ASS234 inhibited the mitochondrial-mediated apoptotic pathway by reducing the levels of cleaved caspases 3 and 9 and the levels of proteolysed PARP [182]. The pyrazolone edaravone reversed the AD-like* in vitro* mitochondrial insults in the transfected N2a/Swe.Δ9 cell model, in which edaravone treatment increased cell viability, attenuating oxidative stress and CytC release and improving MMP; in addition, it diminished apoptotic rate through a decrease in the Bax/Bcl-2 ratio and a suppression of caspase-3 activation [173]. Also, both R(+) and S(−) stereoisomers of pramipexole exerted restorative effects in another A*β*-induced model of AD; they were able to inhibit mitochondrial-mediated apoptotic process by inhibiting caspases activations [209].

Finally, a very recent work has revealed a mitochondrial-targeted protective action for the well-known acetylcholinesterase inhibitor donepezil, which is clinically used for treating AD. Donepezil displayed ameliorative effects on behavioral deficits in APP/PS1 double transgenic mice and enhanced the resistance of their brain mitochondria to the induction of mPTP by calcium ions; such action may be mediated by its lowering effect on mitochondrial A*β* level in brain of treated animals, which was also later confirmed* in vitro* in isolated mitochondria from rat brains. Thus, it avoids the A*β*_1–42_-induced functional decay in mitochondria in such AD model [224].

#### 3.2.2. Endogenous Compounds

Several compounds with endogenous origin have revealed interesting actions on mitochondrial deficiencies of AD. For instance, the hormone melatonin has been largely studied. Dragicevic et al. showed that a pretreatment with melatonin protected cognitive function in an APPsw mice model of AD. A plausible mechanism for the observed effect via mitochondria was tested in isolated mitochondria from mice, proving that melatonin could completely restore mitochondrial respiratory rate, MMP, ROS production, and energy metabolism; other* in vitro* assays suggested a possible implication of c-AMP-dependent phosphodiesterase (PDE) 4 or cGMP-dependent PDE5 in the effects displayed by melatonin [169]. The same group of research previously demonstrated similar actions of melatonin in brain mitochondria isolated from the double transgenic APP/PS1 mice model of AD and determined that melatonin receptor signaling is required for its full effect [170]. Furthermore, melatonin evidenced mitochondrial protective activity in* in vitro* models. Besides acting as an antioxidant activator of mitochondrial aconitase, one of the enzymes of the citric acid cycle that is affected by the oxidative stress in AD [179], melatonin was reported to act as a defensive agent against A*β*-induced cytotoxicity in BV2 microglial cells; it attenuated the cellular apoptosis by activating Bcl-2 antiapoptotic pathways, thus involving higher Bcl-2 expression and reduced Bax mRNA level and caspase-3 activity [197].

The endogenous antioxidant glutathione also acts as a mitochondrial aconitase activator [179] and avoids the A*β*-induced mitochondrial membrane depolarization in human HCN-1A cells [193]. Two mitochondrial metabolites such as the acetyl-L-carnitine and the R-*α*-lipoic acid (LA) have attracted attention in AD-related research. Aliev et al. confirmed that old rats fed with both compounds presented significantly reduced number of damaged mitochondria and increased number of intact mitochondria in hippocampus, thus preventing from mitochondrial decay associated with age and AD [180]. The organosulfur compound LA was able to decrease the mitochondrial-related oxidative stress in fibroblasts from AD patients; at a concentration of 1 mM, LA attenuated AD-type mitochondrial dysfunction generated by the cytochrome oxidase assembly inhibitor N-methylprotoporphyrin [196].

The neuroprotective role of peroxiredoxins (Prdx) 3 y 6, the key mitochondrial antioxidant defense enzymes in detoxifying ROS such as H_2_O_2_, has been evaluated with results suggesting their therapeutic/prophylactic potential to slow and attenuate AD progression and related neuronal death. Rat PC12 cells overexpressing functional Prdx 6 presented diminished A*β*_25–35_-induced mitochondrial apoptotic pathway, expressed by an inhibition of PARP inactivation, caspases 3 and 9 activations, and Bcl-2 and Bax dysregulation [206]. Meanwhile, Chen et al. evidenced the role of Prdx 3 in improving cognition, by using two transgenic mice models of AD (APP and APP/Prdx3 models); Prdx 3 activity was correlated with reduced brain amyloid beta level and production and maintenance of mitochondrial integrity and function, showing reduced mitochondrial DNA oxidation and enhanced activity of mitochondrial complexes I and IV [205]. Nicotinamide, as an endogenous inhibitor of poly-ADP-ribose polymerase-1 (PARP-1), is evidenced to display reduction of oxidative stress in an A*β*_1–42_-induced rat model, and it upregulated mitochondrial function and downregulated mitochondrial apoptosis, lessening Bax levels and increasing Bcl-2 levels [204].

#### 3.2.3. Natural Products

Finally, numerous natural products deserve to be mentioned for their mitochondrial-targeted antioxidant activities. Alkaloid caffeine is proved to exert similar activities to melatonin [169] and in its crude form, resulting from decaffeination of coffee, it reduced memory impairment and A*β*_1–42_ levels in hippocampus of an AD mouse model, the J20 mouse line; crude caffeine maintained mitochondrial function as revealed by increased ATP levels and lower ROS generation and inhibited the apoptosis mediated by mitochondria (reduced caspase-3 activity) [184]. Polyphenol resveratrol, a proven antioxidant with interest in AD [225], when tested in an A*β*-induced N2a mouse cells model, normalized the number of mitochondria and decreased the abnormal expression of peroxiredoxins and mitochondrial structural genes; it also preserved mitochondrial function* in vivo*, by inhibiting CypD expression [200]. Polyphenolic compound tournefolic acid B (TAB) has been isolated from* Tournefortia sarmentosa* Lam. (Boraginaceae) and investigated by Chi et al. on an* in vitro* model of AD, who demonstrated its neuroprotective effect via mitochondrial caspase 8-tBid-cytochrome c pathway. Actually, 50 *μ*M TAB revoked the A*β* protein-induced caspases 8 and 9 activation, significantly reduced the elevation of calcium level in mitochondria, and delayed the release of CytC; reduction in apoptosis was evidenced by an attenuation in mitochondrial tBid elevation, without affecting A*β*-mediated decrease in mitochondrial Bcl-2*α* [214].

Gypenoside XVII (GP-17) is a novel phytoestrogen isolated from* Gynostemma pentaphyllum* or* Panax notoginseng* that, due to its structure, was found to confer neuroprotection against A*β*_25–35_-induced neurotoxicity in PC12 cells via estrogen receptor-dependent activation of PI3K/Akt and Nrf2/ARE/HO-1 pathways and inactivation of PI3K/Akt pathway; regarding mitochondrial failures, pretreatment with GP-17 (10 *μ*M) for 12 h is demonstrated to restore normal MMP and reduced CytC release and the enhanced apoptosis, by inhibiting caspase-3 activation and cleavage [194]. The phytoestrogen genistein also showed its potency to upregulate the mitochondrial Na/K-ATPase activity in human AD brains, which is proved to be disturbed in the early phase of the disease; by ameliorating the energy metabolism in the initiation of AD pathology, genistein is presented as an interesting candidate drug [191].

Wang et al. evaluated the potential activity of acteoside, an antioxidant phenylethanoid glycoside first extracted from* Verbascum sinuatum* and named “verbascoside,” on A*β*_25–35_-induced SHSY5Y cell injury. They concluded that a pretreatment with acteoside for 15 h was effective for inhibiting ROS production and mitochondrial dysfunction and apoptotic pathway; this modulation involved decrease in Bax/Bcl-2 ratio, cytochrome c release, and the cleavage of caspase-3 [181]. Thymoquinone is the bioactive and most abundant constituent of the volatile oil of black seed and has been described as a promising antioxidant in* in vitro* AD models, in part for its restorative action on MMP impairments due to A*β* neurotoxicity [212, 213].

Concerning plant extracts, a green tea leaf extract rich in epicatechin and epigallocatechin gallate, was able to reverse damaging effects of AlCl_3_ in a rat model, suggesting it might be beneficial in AD. It reduced the aluminium neurotoxicity via antioxidant and mitochondrial protective effects, such as an augment in CytC oxidase activity [226].

## Figures and Tables

**Table 1:** Parkinson's disease and mitochondria-targeted protective compounds.

Compound	Class of compound	Parkinson's model	Mechanisms	References
Acetyl-L-carnitine	Quaternary ammonium	*In vitro* human neuroblastoma SK-N-MC cells model of rotenone-induced PD	Maintenance of mitochondrial integrity and function: ↑ mitochondrial biogenesis ↓ ROS generation	[65]

Astaxanthin	Nonprovitamin A carotenoid	MPP(+)-induced mouse model *In vitro* human neuroblastoma SK-N-SH cells model of MPP+-induced PD	Inhibition of the mitochondrial apoptotic pathway: ↑ Bcl-2 ↓ Bax and *α*-synuclein ↓ caspase-3	[81]

		*In vitro* human neuroblastoma SH-SY5Y cells model of 6-OHDA-induced PD	Maintenance of mitochondrial integrity and function: maintain mitochondrial membrane potential; maintain mitochondrial redox activity	[82]
Baicalein	Flavonoid	*In vitro* rat adrenal pheochromocytoma PC12 cells model and isolated rat brain mitochondria of rotenone induced PD	Inhibition of the mitochondrial apoptotic pathway: ↓ caspase-3 and caspase-7 Maintenance of mitochondrial integrity and function: maintain mitochondrial membrane potential ↓ ROS generation ↑ intracellular ATP production	[83]
		*In vitro* human neuroblastoma SH-SY5Y cells model of 6-OHDA-induced PD	Maintenance of mitochondrial integrity and function: maintain mitochondrial membrane potential Inhibition of the mitochondrial apoptotic pathway: ↓ caspase-3 and caspase-9 ↓ p-c-Jun N-terminal kinase (JNK)	[84]

Caffeic acid phenethyl ester	Phenolic compound	6-OHDA-induced rat PD model	Inhibition of the mitochondrial apoptotic pathway: ↓ caspase-3 and caspase-9 ↓ cytochrome c release Maintenance of mitochondrial integrity and function	[85]
		Primary cultures of cerebellar granule neuron 6-OHDA-induced PD	Inhibition of the mitochondrial apoptotic pathway, ↓caspase-3 ↓ cytochrome c release	[86]

CNB-001	Pyrazole derivative of curcumin	MPTP-induced rodent PD model	Maintenance of normal mitochondrial morphology and size	[87]
		*In vitro* human neuroblastoma SK-N-SH cells model of rotenone-induced PD	Inhibition of the mitochondrial apoptotic pathway: ↑ Bcl-2 ↓ Bax ↓ caspase-3 and caspase-9 ↓ cytochrome c release Maintenance of mitochondrial integrity and function: maintain mitochondrial membrane potential	[88]

Curcumin	Polyphenol	PC12 cells mutant A53T *α*-synuclein-induced cell death	Inhibition of the mitochondrial apoptotic pathway: ↓ caspase-3 and caspase-9 ↓ cytochrome c release Maintenance of mitochondrial integrity and function: maintain mitochondrial membrane potential ↓ ROS generation	[89]
		Mouse brain and the 1RB3AN27 (N27) rat dopaminergic neuronal cell line model of buthionine sulfoximine-induced PD	Maintenance of mitochondrial integrity and function: ↑ activities of complex I ↓ oxidative damage to proteins ↑ glutathione	[90]

DL-3-n-butylphthalide (NBP)	Synthetic compound based on L-3-n-butylphthalide	*In vitro* rat adrenal pheochromocytoma (PC12) cells model of MPP(+)-induced PD	Maintenance of mitochondrial integrity and function: maintain mitochondrial membrane potential ↓ ROS generation ↑ glutathione	[91]

Echinacoside	Phenylethanoid glycoside	*In vitro* rat adrenal pheochromocytoma (PC12) cells model of H_2_O_2_-induced PD	Inhibition of the mitochondrial apoptotic pathway	[92]

Edaravone	Pyrazole derivative	Rotenone-induced rat PD model	Inhibition of the mitochondrial apoptotic pathway: ↓ Bax Maintenance of mitochondrial integrity and function: ↓ ROS generation	[93]

Esculetin	Coumarin	MPTP-induced mice PD model	Inhibition of the mitochondrial apoptotic pathway: ↓ caspase-3	[94]

Ginsenoside Re	Panaxatriol saponin	PINK1 null cells	Reversing the deficit in complex IV activity: ↑ LRPPRC, Hsp90, and Hsp60 levels	[95]

Glutamoyl diester of curcumin	Polyphenol	Mouse brain mitochondria induced-peroxynitrite PD	Maintenance of mitochondrial integrity and function: maintain mitochondrial membrane potential ↓ ROS generation	[96]

Harmalol	Beta-carboline	*In vitro* rat adrenal pheochromocytoma (PC12) cells model of SNAP-induced PD	Maintenance of mitochondrial integrity and function: maintain mitochondrial membrane potential ↓ ROS generation	[97]

Harmine	Beta-carboline	*In vitro* rat adrenal pheochromocytoma (PC12) cells model of SNAP-induced PD	Maintenance of mitochondrial integrity and function: maintain mitochondrial membrane potential ↓ ROS generation	[97]

Hesperidin	Polyphenol	*In vitro* human neuroblastoma SK-N-SH cells model of rotenone-induced PD	Inhibition of the mitochondrial apoptotic pathway: ↑ Bcl-2 ↓ Bax ↓ caspase-3 and caspase-9 ↓ cytochrome c release Maintenance of mitochondrial integrity and function: maintain mitochondrial membrane potential ↓ ROS generation ↑ glutathione	[98]

Isoborneol	Monoterpenoid alcohol	*In vitro* human neuroblastoma SH-SY5Y cells model of 6-OHDA-induced PD	Inhibition of the mitochondrial apoptotic pathway: ↑ Bcl-2 ↓ Bax ↓ caspase-3 ↓ cytochrome c release	[99]

Kaempferol	Flavonoid	*In vitro* human neuroblastoma SH-SY5Y cells model and primary neurons of rotenone-induced PD	↑ Autophagy	[100]

*α*-Lipoic acid	Organosulfur compound derived from octanoic acid	*In vitro* rat adrenal pheochromocytoma PC12 cells model of MPP(+)-induced PD	Maintenance of mitochondrial integrity and function: maintain mitochondrial membrane potential ↓ ROS generation	[75]
		Rotenone-induced rat PD model	Maintenance of mitochondrial integrity and function: ↑ mitochondrial complex I activity ↑ glutathione	[76]
		*In vitro* human neuroblastoma SK-N-MC cells model of rotenone-induced PD	Maintenance of mitochondrial integrity and function: ↑ mitochondrial biogenesis ↓ ROS generation	[77]
		*In vitro* rat adrenal pheochromocytoma (PC12) cells model of PD	Maintenance of mitochondrial integrity and function: ↑ mitochondrial complex I activity ↑ glutathione	[78]

Lycopene	Carotenoid	*In vitro* human neuroblastoma SH-SY5Y cells model of MPP(+)-induced PD	Maintenance of mitochondrial integrity and function: maintain mitochondrial membrane potential ↓ ROS generation ↓ lipid peroxidation ↑ intracellular ATP production ↑ mitochondrial DNA copy numbers and mitochondrial RNA transcript levels	[101]
		Rotenone-induced rat PD model	Inhibition of the mitochondrial apoptotic pathway: ↓ cytochrome c release Maintenance of mitochondrial integrity and function: ↓ lipid peroxidation ↑ SOD activity ↑ glutathione	[102]

		MPTP-induced mice PD model	Maintenance of mitochondrial integrity and function: maintain mitochondrial membrane potential ↑ antioxidant enzyme levels ↑ intracellular ATP production	[67]
		Rotenone-induced rat PD model	Maintenance of mitochondrial integrity and function: ↓ ROS generation ↑ glutathione ↑ SOD and catalase activities	[68]
Melatonin	Hormone	Rotenone-induced isolated rat brain mitochondria PD model	Maintenance of mitochondrial integrity and function: ↓ Ca^2+^ levels ↓ ROS generation	[69]
		6-OHDA-induced rat PD model	Maintenance of mitochondrial integrity and function: ↑ mitochondrial complex I activity	[70]
		MPP(+)-induced isolated rat liver mitochondria and striatal synaptosomes PD model	Maintenance of mitochondrial integrity and function: ↑ mitochondrial complex I activity	[71]

MitoQ (mitoquinone mesylate)	—	*In vitro* human neuroblastoma SH-SY5Y cells model of 6-OHDA-induced PD	Inhibition of the mitochondrial apoptotic pathway: ↓ Bax ↓ Drp1	[73]

N-Acetylcysteine	Amino acid derivative	H_2_O_2_ and toxic quinones derived from dopamine-induced rat PD model	Maintenance of mitochondrial integrity and function: ↑ mitochondrial respiratory activity ↑ Na+, K+-ATPase activity	[103]

Neuropeptide cocaine and amphetamine regulated transcript (CART)	Peptide	*In vitro* human neuroblastoma SH-SY5Y, HEK293 cells, and cultures of cortical and hippocampal neurons of H_2_O_2_-induced PD	Maintenance of mitochondrial integrity and function: protection of mitochondrial DNA (mtDNA), cellular proteins, and lipids	[78]

Osthole	Coumarin	*In vitro* rat adrenal pheochromocytoma (PC12) cells model of MPP(+)-induced PD	Inhibition of the mitochondrial apoptotic pathway: ↑ Bcl-2 ↓ Bax ↓ caspase-3 ↓ cytochrome c release	[104]

Piperine	Alkaloid	6-OHDA-induced rat PD model	Inhibition of the mitochondrial apoptotic pathway: ↑ Bcl-2 ↓ Bax ↓ caspase-3 and caspase-9 ↓ cytochrome c release	[105]

Polyhydroxylated fullerene derivative C(60)(OH)(24)	—	*In vitro* human neuroblastoma cells model of MPP(+)-induced PD	Maintenance of mitochondrial integrity and function: maintain mitochondrial membrane potential ↓ ROS generation ↑ activities of complexes I and II ↓ oxidative damage to DNA and proteins	[106]

Protocatechuic acid	Polyphenol	*In vitro* rat adrenal pheochromocytoma (PC12) cells model of MPP(+)-induced PD	Inhibition of the mitochondrial apoptotic pathway: ↑ Bcl-2 ↓ caspase-3 Maintenance of mitochondrial integrity and function: maintain mitochondrial membrane potential ↓ ROS generation ↑ glutathione	[107]

Pyruvate	Organic acid	*In vitro* human neuroblastoma SK-N-SH cells model of H_2_O_2_-induced PD	Maintenance of mitochondrial integrity and function: maintain mitochondrial membrane potential ↓ ROS generation	[79]
		*In vitro* rat adrenal pheochromocytoma (PC12) cells model of dopamine-induced PD	Maintenance of mitochondrial integrity and function: maintain mitochondrial membrane potential ↓ ROS generation ↓ p53 ↓ nuclear translocation of NF-kappaB Inhibition of the mitochondrial apoptotic pathway	[80]

Quercetin	Bioflavonoid	Rotenone-induced rat PD model	Maintenance of mitochondrial integrity and function: ↓ ROS generation maintain mitochondrial membrane potential	[108]
		*In vitro* glial-neuronal system model of MPP(+)-induced PD	Maintenance of mitochondrial integrity and function: ↓ inducible nitric oxide synthase protein expression ↓ superoxide radicals	[109]

Rapamycin	Macrolide	6-OHDA-induced rat PD model	Inhibition of the mitochondrial apoptotic pathway: ↑ Bcl-2 ↓ Bax ↓ caspase-9 ↓ cytochrome c release Maintenance of mitochondrial integrity and function: ↓ lipid peroxidation ↑ SOD and GSH-PX	[110]

Resveratrol	Polyphenol	*In vitro* primary fibroblasts cultures from patients with parkin mutations (PARK2)	Maintenance of mitochondrial integrity and function: ↑ complex I activity ↑ citrate synthase activity ↑ basal oxygen consumption ↑ mitochondrial ATP production ↓ lactate content	[111]

Rosmarinic acid	Polyphenol	*In vitro* MES23.5 dopaminergic cells model of 6-OHDA-induced PD.	Maintenance of mitochondrial integrity and function: maintain mitochondrial membrane potential ↓ ROS generation	[112]

Salvianic acid A	Polyphenol	*In vitro* human neuroblastoma SH-SY5Y cells model of MPP(+)-induced PD	Inhibition of the mitochondrial apoptotic pathway: ↑ Bcl-2 ↓ Bax	[113]

Salvianolic acid B	Polyphenol	*In vitro* human neuroblastoma SH-SY5Y cells model of 6-OHDA-induced PD	Inhibition of the mitochondrial apoptotic pathway: ↑ Bcl-2 ↓ Bax ↓ caspase-3 ↓ cytochrome c release	[114]

Sesamin	Lignan	*In vitro* glial-neuronal system model of MPP(+)-induced PD	Maintenance of mitochondrial integrity and function: ↓ inducible nitric oxide synthase protein expression ↓ superoxide radicals	[22]

Tetramethylpyrazine	Pyrazine	MPTP-induced rat PD model	Inhibition of the mitochondrial apoptotic pathway: ↑ Bcl-2 ↓ Bax ↓ caspase-3 ↓ cytochrome c release	[115]

Tyrosol	Phenolic compound	MPP(+)-induced CATH.a cells PD model	Maintenance of mitochondrial integrity and function: maintain mitochondrial membrane potential; maintain intracellular ATP production Inhibition of the mitochondrial apoptotic pathway: (i) activation of PI3K/Akt signalling pathway	[116]

Umbelliferone	Coumarin	MPTP-induced mice PD model	Inhibition of the mitochondrial apoptotic pathway: ↓ caspase-3	[94]

Ursodeoxycholic acid	Secondary bile acids	*In vitro* human neuroblastoma SH-SY5Y cells model of SNP-induced PD	Inhibition of the mitochondrial apoptotic pathway: ↑ Bcl-2 ↓ Bax ↓ caspase-3, caspase-7, and caspase-9 ↓ cytochrome c release Maintenance of mitochondrial integrity and function: maintain mitochondrial membrane potential ↓ ROS generation ↑ glutathione	[74]

Xyloketal B	Triterpenoid	*In vitro* rat adrenal pheochromocytoma (PC12) cells model and *Caenorhabditis elegans* of MPP(+)-induced PD	Maintenance of mitochondrial integrity and function: maintain mitochondrial membrane potential ↓ ROS generation ↑ glutathione	[117]

**Table 2:** Alzheimer's disease and mitochondria-targeted protective compounds.

Compound	Class of compound	AD model	Mechanism/effect	References
17-*β*-Estradiol	Estrogen	*In vitro *isolated mitochondria from *postmortem* human AD and SFAD brains	Activation of mitochondrial aconitase	[179]

Acetyl-L-carnitine	Amino acid	*In vivo* F344 rat model	Restoration of intact mitochondrial morphology and prevention from age-related mitochondrial decay	[180]

Acteoside	Glycoside	*In vitro* Aβ_25–35_-induced SH-SY5Y human neuroblastoma cells model	Maintenance of mitochondrial integrity and function: ↓ ROS production ↓ MMP loss ↓ CytC release Inhibition of the mitochondrial apoptotic pathway: ↓ Bax/Bcl-2 ratio ↓ caspase-3 cleavage	[181]

ASS234	Amine with complex structure	*In vitro* A*β*_1–42_-induced SH-SY5Y human neuroblastoma cells model	Inhibition of the mitochondrial apoptotic pathway: ↓ levels of cleaved caspases 3 and 9 ↓ levels of proteolysed PARP	[182]

Caffeine	Alkaloid	*In vivo* APPsw mice model	Maintenance of mitochondrial integrity and function: restoration of mitochondrial respiratory rate, maintenance of MMP ↓ ROS production ↑ ATP levels	[169]

Colostrinin	Polypeptide	*In vitro* A*β*-induced human SH-SY5Y and rat PC12 cells model	Maintenance of mitochondrial integrity and function: ↓ mitochondrial H_2_O_2_ production	[183]

Crude caffeine	Alkaloid	*In vitro* primary neurons from J20 mouse line	Maintenance of mitochondrial integrity and function: ↑ ATP levels ↓ ROS production Inhibition of the mitochondrial apoptotic pathway: ↓ caspase-3 activity	[184]

Cyclosporin A	—	*In vitro* isolated cortical mitochondria from mAPP-Ppif^−/−^transgenic mice	Maintenance of mitochondrial integrity and function: inhibition of mPTP opening ↑ Ca^2+^ buffering capacity	[185]

D609	Tricyclodecan-9-yl-xanthogenate	*In vitro* A*β*_1–42_-induced isolated brain mitochondria model (from gerbils)	Maintenance of mitochondrial integrity and function: ↓ levels of protein carbonyls ↓ levels of protein-bound HNE ↓ levels of 3-NT ↓ CytC release Maintenance of GSH/GSSG ratio ↑ GST, GPx, and GR activities	[186]

DAPT	Complex structure of butyl ester	*In vitro* N2a/APP695 and N2a/APPswe cells models	Maintenance of mitochondrial integrity and function: stabilization of normal MMP and ATP levels	[187]

Dimebon	Tetrahydrocarboline	*In vitro* A*β*_25–35_-exposed isolated rat brain mitochondria	Maintenance of mitochondrial integrity and function: inhibition of mPTP opening	[188]

Edaravone	Pyrazolinone	*In vitro* N2a/Swe.Δ9 cells model	Maintenance of mitochondrial integrity and function, ↑ MMP ↓ ROS production ↓ CytC release Inhibition of the mitochondrial apoptotic pathway: ↓ Bax/Bcl2 ratio ↓ Caspase-3 activation	[173]

Ergothioneine	2-Mercaptohistidine trimethylbetaine	*In vitro* A*β*_25–35_-induced PC12 rat pheochromocytoma cells model	Maintenance of mitochondrial integrity and function: ↓ MMP loss Inhibition of the mitochondrial apoptotic pathway, ↓ ratio Bax/Bcl-X_L_ ↓ caspase-3 activation	[189]

Gelsolin	Peptide	*In vitro* A*β*_1–42_-induced rat epithelial cells model	Maintenance of mitochondrial integrity and function: increase in mitochondrial complex IV activity	[190]

Genistein	Phytoestrogen	Mitochondrial fraction of *postmortem* human AD brains	↑ mitochondrial Na/K-ATPase activity	[191]

GLP-1 (9-36)^amide^	Peptide	*In vitro* slices of hippocampus from APP/PS1 mice	Maintenance of mitochondrial integrity and function: ↓ abnormal mitochondrial SO levels	[192]

Glutathione	Tripeptide	*In vitro* A*β*-induced human HCN-1A cells model	Maintenance of mitochondrial integrity and function: ↓ mitochondrial membrane depolarization	[193]
		*In vitro *isolated mitochondria from p*ostmortem* human AD and SFAD brains	Activation of mitochondrial aconitase	[179]

Gypenoside XVII	Phytoestrogen	*In vitro *A*β*_25–35_-induced PC12 cells model	Maintenance of mitochondrial integrity and function: restoration of normal MMP ↓ CytC release Inhibition of the mitochondrial apoptotic pathway: ↓ caspase-3 activation and cleavage ↓ PARP cleavage	[194]

JHX-4, HK-2, and HK-4	—	*In vitro* A*β*-induced human SH-SY5Y cells model	Prevention from MnCl_2_-induced loss of mitochondrial activity	[195]

Lipoic acid	Organosulfur compound derived from octanoic acid	*In vivo* F344 rat model	Restoration of intact mitochondrial morphology and prevention from age-related mitochondrial decay	[180]
		*In vitro *fibroblasts from AD patients	Maintenance of mitochondrial integrity and function: ↓ NMP-induced mitochondrial oxidative stress Inhibition of the mitochondrial apoptotic pathway: ↓ Bax and caspase-9 levels	[196]

		*In vitro* A*β*_25–35_-induced mouse microglial BV2 cells model	Inhibition of the mitochondrial apoptotic pathway: activation of Bcl-2 antiapoptotic pathways ↓ Bax mRNA level ↑ Bcl-2 expression ↓ caspase-3 activity	[197]
		*In vitro* A*β*_25–35_-induced rat hippocampal neurons model	Maintenance of mitochondrial integrity and function: restoration of MMP loss attenuation of respiratory chain complexes ↑ ATP levels	[198]
Melatonin	Hormone	*In vivo* APP/PS1 transgenic mice	Maintenance of mitochondrial integrity and function: restoration of mitochondrial respiratory rates ↑ MMP ↑ ATP levels	[170]
		*In vivo* APPsw mice model	Maintenance of mitochondrial integrity and function, restoration of mitochondrial respiratory rate maintenance of MMP ↓ ROS production ↑ ATP levels	[169]
		*In vitro *isolated mitochondria from *postmortem* human AD and SFAD brains	Activation of mitochondrial aconitase	[179]
		*In vivo* APP transgenic mice model	Maintenance of mitochondrial integrity and function, ↑ SOD activity Inhibition of the mitochondrial apoptotic pathway, ↓ Bax, caspase-3, and Par-4 expressions	[168]

Methylene blue	Phenothiazine	*In vivo* C57BL/6 mice	Maintenance of mitochondrial integrity and function, ↑ mitochondrial complex IV (CytC oxidase) activity ↓ MAO activity	[199]

		*In vitro *A*β*-induced mouse neuroblastoma N2a cells model	Maintenance of mitochondrial integrity and function, ↓ abnormal expression of peroxiredoxins and mitochondrial structural genes normalization in mitochondria number	[200]
Mito Q	Methanesulfonate	*In vivo* A*β*PP transgenic mice	Maintenance of mitochondrial integrity and function: ↓ CypD expression	
		*In vivo* 3xTg-AD mice	Maintenance of mitochondrial integrity and function: prevention of MMP loss Inhibition of the mitochondrial apoptotic pathway: blockage of caspases-3/7 activation	[171]
		*In vivo Caenorhabditis elegans *model overexpressing human A*β* peptides	Maintenance of mitochondrial integrity and function, ↓ depletion of mitochondrial lipid cardiolipin Protection of complexes IV and I of the ETC	[201]

Mitochondrial division inhibitor 1 (Mdivi-1)	Quinazolinone	*In vitro* A*β*-induced mouse BV-2 cells and primary microglial cells model	Maintenance of mitochondrial integrity and function: ↓ abnormal mitochondrial fission ↓ MMP loss ↓ CytC release Inhibition of the mitochondrial apoptotic pathway: ↓ caspase-3 activation	[182]
		*In vitro* cybrid cells model (mtDNA-depleted neuronal SH5Y5Y cells transfected with platelet mitochondria from AD patient)	Maintenance of mitochondrial integrity and function: ↓ mitochondrial ROS production ↑ MMP ↑ mitochondrial length and density ↑ CytC oxidase activity ↑ SOD activity ↑ ATP levels ↓ impaired mitochondrial fission and fusion dynamics	[174]

N-Acetyl cysteine	Amino acid derivative	*In vitroβ*-amyloid-induced human HCN-1A cells model	Maintenance of mitochondrial integrity and function: ↓ mitochondrial membrane depolarization	[193]
		*In vivo* APP/PS-1 mice	Amelioration of energy levels and mitochondrial-related proteins	[202]
		*In vitro *fibroblasts from AD patients	Maintenance of mitochondrial integrity and function: ↓ NMP-induced mitochondrial oxidative stress Inhibition of the mitochondrial apoptotic pathway: ↓ Bax and caspase-9 levels	[196]

Neuregulin-1	Peptide	*In vitro* model of SH-SY5Y human neuroblastoma cells transfected with C-terminal fragments of APP	Maintenance of mitochondrial integrity and function: ↓ MMP loss Inhibition of the mitochondrial apoptotic pathway: ↑ Bcl-2 expression	[203]

Nicotinamide	Amide	*In vivo* A*β*_1–42_-induced rat model	Upregulation of mitochondrial function Inhibition of the mitochondrial apoptotic pathway: ↑ Bcl-2 levels ↓ Bax levels	[204]

Peroxiredoxin 3	Enzyme	*In vivo* APP transgenic mice (Tg2576) and APP/Prdx3 transgenic mice models	Maintenance of mitochondrial integrity and function: ↓ mitochondrial DNA oxidation ↑ activity of mitochondrial complexes I and IV	[205]

Peroxiredoxin 6	Enzyme	*In vitro *A*β*_25–35_-induced PC12 cells model	Inhibition of the mitochondrial apoptotic pathway: ↓ caspases 3 and 9 activation ↓ PARP activation ↓ Bcl-2 and Bax dysregulations	[206]

Probucol	—	*In vitro* cybrid cells model (mtDNA-depleted neuronal SH5Y5Y cells transfected with platelet mitochondria from AD patient)	Maintenance of mitochondrial integrity and function: ↓ mitochondrial ROS production ↑ MMP ↑ mitochondrial length and density ↑ CytC oxidase activity ↑ SOD activity ↑ ATP levels ↓ impaired mitochondrial fission and fusion dynamics	[174]

Puerarin	Phytoestrogen	*In vivo* A*β*_1–42_-induced rat model	Inhibition of the mitochondrial apoptotic pathway: ↓ caspase-9 activity and mRNA levels	[207]
		*In vitro* cybrid cells model (mtDNA-depleted neuronal SH-5Y5Y cells transfected with platelet mitochondria from AD patient)	Inhibition of the mitochondrial apoptotic pathway: ↑ Bcl-2 levels ↓ Bax expression ↓ caspase-3 activity	[208]

R(+) and S(−) pramipexoles	—	*In vitro* A*β*_25–35_-induced SH-SY5Y human neuroblastoma cells model	Maintenance of mitochondrial integrity and function: inhibition of mPTP opening Inhibition of the mitochondrial apoptotic pathway: ↓ caspases 3 and 9 activations	[209]

Resistin	Adipokine protein	*In vitro* mouse N2a/Swe.Δ9 cells (N2a cells transfected with Sw-APP mutant and presenilin exon 9 deletion mutant)	Maintenance of mitochondrial integrity and function: ↑ ATP levels ↑ MMP Inhibition of the mitochondrial apoptotic pathway: ↓ Bax/Bcl2 ratio ↓ CytC release ↓ caspase-3 activation	[210]

Resveratrol	Polyphenol	*In vitro *A*β*-induced mouse neuroblastoma N2a cells model	Maintenance of mitochondrial integrity and function: ↓ abnormal expression of peroxiredoxins and mitochondrial structural genes Normalization in mitochondria number	[200]
		*In vivo* A*β*PP transgenic mice	Maintenance of mitochondrial integrity and function: ↓ CypD expression	

Salicylate Sulindac sulfide Indomethacin Ibuprofen R-flurbiprofen	NSAIDs	*In vitro* Aβ_1–42_- and A*β*_25–35_-induced rat cerebellar granule cells and cortical neurons model	Maintenance of mitochondrial integrity and function: ↑ MMP ↓ mitochondrial Ca^2+^ overload ↓ CytC release	[172]

SS31	Peptide	*In vitro* A*β*_25–35_-induced C57BL/6 mice hippocampal neurons model	Maintenance of mitochondrial integrity and function: ↓ levels of mitochondrial fission proteins (Drp1, Fis1), matrix protein, and CypD ↑ number of healthy and intact mitochondria Restoration of mitochondrial transport	[162]
		*In vitro *A*β*-induced mouse neuroblastoma N2a cells model	Maintenance of mitochondrial integrity and function: ↓ abnormal expression of peroxiredoxins and mitochondrial structural genes Normalization in mitochondria number	[200]
		*In vivo* A*β*PP transgenic mice	Maintenance of mitochondrial integrity and function: ↓ CypD expression	
		*In vitro* Tg2576 mice primary neurons	Maintenance of mitochondrial integrity and function: restoration of mitochondrial transport ↓ percentage of defective mitochondria	[211]

Thymoquinone	Benzoquinone	*In vitro* A*β*_25–35_-induced PC12 cells model	Maintenance of mitochondrial integrity and function: ↑ MMP	[212]
		*In vitro* A*β*_1–42_-induced primary rat neurons model	Maintenance of mitochondrial integrity and function: ↑ MMP	[213]

Tournefolic acid B	Polyphenol	*In vitro β*_25–35_-induced rat cortical neurons model	Maintenance of mitochondrial integrity and function: ↓ mitochondrial Ca^2+^levels delay in CytC release Inhibition of the mitochondrial apoptotic pathway: ↓ tBid levels	[214]

Trolox	Analog of vitamin E	*In vitro *dentate granule cells from transgenic mouse AD model (Tg2576)	Maintenance of mitochondrial integrity and function: scavenging of mitochondrial SO restoration of Ca^2+^ clearance ↑ MMP	[215]

UPF1 and UPF17	Peptides	Mitochondrial fraction of *postmortem* human AD brains	↑ Mitochondrial Mn-SOD activity	[191]

Vitamin C	Vitamin	*In vitro* A*β*-induced human HCN-1A cells model	Maintenance of mitochondrial integrity and function: ↓ mitochondrial membrane depolarization	[193]
		*In vivo* 5XFAD Knockout-transgenic mice model	Maintenance of mitochondrial integrity and function: prevention of abnormal mitochondrial morphology	[216]

Wy-14.463 (peroxisome proliferator)	—	*In vitro* A*β*_1–40_-induced primary rat hippocampal neurons model	Maintenance of mitochondrial integrity and function: ↑ MMP	[217]

Zeolite	—	*In vitro * H_2_O_2_-induced SH-SY5Y cells model	Maintenance of mitochondrial integrity and function: ↓ mitochondrial ROS production	[218]
		*In vivo* APPswePS1dE9 transgenic mice model	Significant increase in SOD activity	

ETC: electron transport chain; MMP: mitochondrial membrane potential; CytC: cytochrome C; SO: superoxide; SOD: superoxide dismutase; SwAPP: Swedish amyloid precursor protein; ROS: reactive oxygen species; 4HNE: 4-hydroxy-2-nonenal; AChE: acetylcholine esterase; GSH: glutathione; GR: glutathione reductase; GPx: glutathione peroxidase; SFAD: Swedish Familial Alzheimer's disease; MAO: monoamine oxidase; tBid: BH3 interacting domain death agonist; Bak: Bcl-2 agonist killer 1; mPTP: mitochondrial permeability transition pore; CypD: cyclosporine D; NMP: N-methyl protoporphyrin; SP-04: dimethyl-carbamic acid 2,3-bis-dimethylcarbamoyloxy-6-(4-ethyl-piperazine-1-carbonyl)-phenyl ester; SP-04m: 4-(4-ethyl-piperazin-1-yl)-1-(2,3,4-trihydroxy-phenyl)-butan-1-one; CCCP: carbonyl cyanide 3-chlorophenylhydrazone; FCCP: carbonyl cyanide p-trifluoromethoxy-phenylhydrazone; PAO: phenylarsine oxide; PARP: poly-ADP-ribosyl polymerase; mtDNA: mitochondrial DNA; Par-4: prostate apoptosis response-4; 3-NT: 3-nitrotyrosine; GSH: reduced glutathione; GSSG: oxidized glutathione; GST: glutathione-S-transferase; GPx: glutathione peroxidase; GR: glutathione reductase; ASS234: N-((5-(3-(1-benzylpiperidin-4-yl)propoxy)-1-methyl-1H-indol-2-yl)methyl)-N-methylprop-2-yn-1-amine; APP: amyloid precursor protein; A*β*: *β* amyloid; NSAIDs: nonsteroidal anti-inflammatory drugs.
